# Supplementary material for: A phase 2/3 study of S-217622 in participants with SARS-CoV-2 infection (Phase 3 part)
Source: Medicine (Baltimore). 2023 Feb 22;102(8):e33024. doi: 10.1097/MD.0000000000033024 (PMC9949372; doi:10.1097/MD.0000000000033024)
Supplement: Supplementary file 1 [file medi-102-e33024-s001.pdf]

Supplemental Table 1. COVID-19 symptom score.

S. No.	Symptom[a]	Response[b]
**1.**	Low energy or tiredness	0: None<br>1: Mild<br>2: Moderate<br>3: Severe
**2.**	Muscle or body aches	
**3.**	Headache	
**4.**	Chills or shivering	
**5.**	Feeling hot or feverish	
**6.**	Stuffy or runny nose	
**7.**	Sore throat	
**8.**	Cough	
**9.**	Shortness of breath (difficulty breathing)	
**10.**	Nausea (feeling like you wanted to throw up)	
**11.**	Vomiting (throw up)	
**12.**	Diarrhea (loose or watery stools)	
**13**	Rate your sense of smell in the last 24 hours	0: My sense of smell is **the same as usual**<br>1: My sense of smell is **less than usual**<br>2: I have **no** sense of smell
**14.**	Rate your sense of taste in the last 24 hours	0: My sense of taste is **the same as usual**<br>1: My sense of taste is **less than usual**<br>2: I have **no** sense of taste

[a]For items 1–10, sample item wording could be: "What was the severity of your symptom at its worst over the last 24 hours?"

[b]Score values are included in the table for ease of reference. The FDA cautions against including the score values within the response options presented to patients to avoid confusion.

COVID-19 = coronavirus disease 2019, FDA = Food and Drug Administration.
