# Supplementary material for: A phase 2/3 study of S-217622 in participants with SARS-CoV-2 infection (Phase 3 part)
Source: Medicine (Baltimore). 2023 Feb 22;102(8):e33024. doi: 10.1097/MD.0000000000033024 (PMC9949372; doi:10.1097/MD.0000000000033024)
Supplement: Supplementary file 2 [file medi-102-e33024-s002.pdf]

Supplemental Table 2. Additional eligibility criteria and endpoints.

<table>
<tr><th colspan="2">Additional eligibility criteria</th></tr>
<tr><th>Inclusion criteria</th><th>Exclusion criteria</th></tr>
<tr><td>• Both male and female patients will be eligible.
• Male patients must not donate sperm during the study intervention period and for at least 10 days after the administration of the last study intervention.
• Male patients must practice abstinence or use contraception during the study period and for at least 10 days after the administration of the last study intervention.
• Female patients must either not be of child-bearing potential or be pregnant or breastfeeding.
• Women of child-bearing potential must use contraception/barriers during the study intervention period and for at least 10 days after the administration of the last study intervention, must not donate eggs during the study, and must have a negative result on a pregnancy test (urine or serum) within 24 hours prior to the first dose of the study intervention.</td><td>• Patients have previously received ensitrelvir.
• Patients have donated ≥400 mL of blood in the 12 weeks or ≥200 mL blood in the 4 weeks prior to providing informed consent/assent.
• Patients have been exposed to ≥4 new chemical entities within 12 months prior to dosing.
• Patients are enrolled or have participated in any other clinical study involving an interventional drug or any other medical research within 28 days prior to providing informed consent.
• Patients have difficulty in entering details into the patient diary properly due to cognitive decline, have a history of drug abuse, or are considered ineligible for the study by the investigator or sub-investigator due to sensitivity to any of the study interventions or their components thereof, have known allergic reactions to any drug, or have a history of other allergies (except for seasonal allergies), or for any other reason.</td></tr>
<tr><th colspan="2">Additional endpoints</th></tr>
<tr><td colspan="2">• Time to resolution of fever (<37°C)
• Time to sustained negative SARS-CoV-2 viral titer
• Proportion of patients with positive SARS-CoV-2 viral titer at each time point
• SARS-CoV-2 viral titer at each time point
• Relative change rate from baseline in SARS-CoV-2 viral titer at each time point
• AUC of change in SARS-CoV-2 viral titer
• Proportion of patients with positive RT-PCR result at each time point
• Amount of SARS-CoV-2 viral RNA at each time point
• Relative change rate from baseline in the amount of SARS-CoV-2 viral RNA at each time point
• AUC of change in the amount of SARS-CoV-2 viral RNA</td></tr>
</table>

• Time to score of ≥1, ≥2, ≥3, ≥4, ≥5, ≥6, and 7 on the 8-point ordinal scale<br>• SpO_2 at each time point<br>• Change from baseline in EQ-5D-5L<br>• Ensitrelvir plasma concentration (Days 2, 6)

AUC = area under the curve, EQ-5D-5L = EuroQol 5 dimension 5 level, RNA = ribonucleic acid, RT-PCR = reverse transcription-polymerase chain reaction, SARS-CoV-2 = severe acute respiratory syndrome coronavirus 2, SpO_2 = saturation of percutaneous oxygen.
