# Supplementary material for: A phase 2/3 study of S-217622 in participants with SARS-CoV-2 infection (Phase 3 part)
Source: Medicine (Baltimore). 2023 Feb 22;102(8):e33024. doi: 10.1097/MD.0000000000033024 (PMC9949372; doi:10.1097/MD.0000000000033024)
Supplement: Supplementary file 4 [file medi-102-e33024-s004.pdf]

Supplemental Table 3. The 8-point ordinal scale for assessing symptom severity.

**Descriptor**	**Score**
Asymptomatic	0
Symptomatic, no limitation of activities	1
Symptomatic, limitation of activities	2
Hospitalized, no oxygen therapy	3
Hospitalized, with oxygen therapy (<5 L/min)	4
Hospitalized, with oxygen therapy (≥5 L/min)	5
Hospitalized, with ventilation	6
Death	7
