# Supplementary material for: A phase 2/3 study of S-217622 in participants with SARS-CoV-2 infection (Phase 3 part)
Source: Medicine (Baltimore). 2023 Feb 22;102(8):e33024. doi: 10.1097/MD.0000000000033024 (PMC9949372; doi:10.1097/MD.0000000000033024)
Supplement: Supplementary file 5 [file medi-102-e33024-s005.pdf]

Supplemental Table 4. List of safety laboratory assessments.

S. No.	Laboratory assessments	Parameters
**1.**	Hematology	Platelet count, red blood cell count, hemoglobin, hematocrit, red blood cell index (MCV, MCH, reticulocyte count), white blood cell count, differential white blood count (neutrophils, lymphocytes, monocytes, eosinophils, and basophils)
**2.**	Blood chemistry[a]	AST, ALT, total bilirubin, direct bilirubin, GGT, LDH, ALP, uric acid, cholinesterase, total protein, albumin, BUN, serum creatinine, sodium, potassium, chloride, calcium, phosphorus, total cholesterol, blood glucose (fasting), HDL-C, LDL-C, TG, CRP, CK, iron, ferritin, UIBC
**3.**	Coagulation test	PT/INR, APTT, fibrinogen
**4.**	Serology	IgG, IgM, haptoglobin
**5.**	Urinalysis	pH, glucose, protein (qualitative), occult blood, ketones, bilirubin, urobilinogen
**6.**	Other	IL-6, IFN-λ3, TARC (CCL17), D-dimer, procalcitonin, KL-6

[a]All events of ALT ≥3×ULN and bilirubin ≥2×ULN (>35% direct bilirubin) or ALT ≥3×ULN and INR >1.5, if INR measured which may indicate severe liver injury, must be reported as an SAE.

ALP = alkaline phosphatase, ALT = alanine aminotransferase, APTT = activated partial thromboplastin time, AST = aspartate aminotransferase, BUN = blood urea nitrogen, CCL = C-C motif ligand, CK = creatine kinase, CRP = C-reactive protein, GGT = gamma-glutamyltransferase, HDL-C = high-density lipoprotein cholesterol, IFN = interferon, IgG = immunoglobulin G, IgM = immunoglobulin M, IL = interleukin, KL-6 = Krebs von den Lungen 6, LDH = lactate dehydrogenase, LDL-C = low-density lipoprotein cholesterol, MCH = mean corpuscular hemoglobin, MCV = mean corpuscular volume, PT/INR = prothrombin time and international normalized ratio, SAE = serious adverse event, TARC = thymus and activation-regulated chemokine, TG = triglycerides, UIBC = unsaturated iron binding capacity, ULN = upper limit of normal.
